# The future of cow’s milk allergy – milk ladders in IgE-mediated food allergy

**DOI:** 10.3389/fnut.2024.1371772

**Published:** 2024-02-28

**Authors:** Allison Hicks, David Fleischer, Carina Venter

**Affiliations:** Section of Pediatric Allergy and Immunology, Children’s Hospital Colorado, University of Colorado School of Medicine, Aurora, CO, United States

**Keywords:** food allergy, cow’s milk allergy, nutrition, food ladders, pediatric

## Abstract

Cow’s milk allergy (CMA) is one of the most common and complex presentations of allergy in early childhood. CMA can present as IgE and non-IgE mediated forms of food allergy. Non-IgE mediated CMA includes food protein-induced enterocolitis syndrome (FPIES), eosinophilic gastrointestinal disorders (EGIDs), and food protein-induced proctocolitis (FPIAP). There are recent guidelines addressing CMA diagnosis, management, and treatment. Each of these guidelines have their own strengths and limitations. To best manage CMA, individualized avoidance advice should be given. Cow’s milk (CM) can be replaced in the diet by using hypoallergenic formulas or plant-based milk, depending on factors such as the child’s age and their current food intake. Oral and epicutaneous immunotherapy is used to increase tolerance in children with CMA but is not without risk, and the long-term outcome of sustained unresponsiveness is still unclear. The allergenicity of CM proteins are affected differently by different forms of heating, leading to the use of baked milk or milk ladders in the management of CMA, most likely the most promising option for future management and treatment of CMA. Future management of children with CMA will also include discussion around the immunomodulatory potential of the child’s dietary intake.

## Introduction

Cow’s milk allergy (CMA) is among the most common food allergies in children, with, for example, a prevalence of 1.8% in children aged 1 to 5 in the United States (1). CMA is divided into IgE mediated and non-IgE mediated CMA, although the European Academy of Asthma, Allergy and Clinical Immunology (EAACI) has recently suggested a more complex nomenclature, focusing on the underlying immunology (2). Diagnosis of CMA includes taking a clinical history, deciding on appropriate testing, followed by an oral food challenge (OFC) for IgE-mediated CMA or a period of avoidance followed by reintroduction/OFC for non-IgE mediated cow’s milk allergies (FPIES, Eosinophilic Esophagitis [EoE], FPIAP). According to the recent EAACI guidelines “A medically supervised oral food challenge (OFC) is recommended to confirm or exclude food allergy in patients with an unclear diagnosis despite IgE-sensitization tests (high certainty of evidence)” (3). Current management strategies include individualized avoidance of foods containing cow’s milk (CM), and precautionary advisory labelling. Depending on age, a hypoallergenic formula or plant-based substitute is recommended (4). For IgE-mediated CMA, emergency medications including epinephrine are used to treat anaphylaxis to CM. For IgE-mediated CMA, oral and epicutaneous immunotherapy can be used to increase tolerance in children but is not without risk, and the long-term outcome of sustained unresponsiveness is still unclear (5) (Waserman et al., 2023, Submitted)^1^. Many guidelines have recently been published to improve the diagnosis, management, and treatment of CMA (3, 6–7) (See footnote 1).

Prognosis is favorable for all types of CMA. For IgE-mediated CMA, approximately 80% of children outgrow their allergy by age 6 (8). It has been known for some time that children with CMA can often tolerate baked forms of the food, especially when combined with a flour matrix (9, 10) while still demonstrating symptoms to unbaked forms, with some studies reporting as much as 70% of CMA children tolerating BM (11–15).

At this time it remains a standard recommendation to offer an observed OFC to baked milk (BM) followed by continued home ingestion of similar products (16–18). However, even after tolerance of BM in an OFC setting, a significant number of patients continue to avoid BM. Dunlop et al. reported 28% of patients sent home with a plan for BM ingestion were avoiding CM in all forms 2 to 7 years later (19). Hicks et al. have recently conducted an international survey of children who had successfully passed a BM challenge and were instructed to introduce BM at home. It was indicated that 88% of participants were instructed to eat any BM-containing food or suitable commercial option. Still, only 27% were given suitable recipes, and the majority received only 1–2 recipes, demonstrating first-hand the need for improved, standardized guidance for families regarding the home introduction of BM (20). For non-IgE mediated CMA, the use of BM in the management of FPIES and EoE have been poorly studied, with two studies indicating that BM foods may be suitable in these patient populations (21, 22). An alternative approach is an at-home food “ladder” approach, used safely in milder forms of non-IgE mediated CMA such as FPIAP (23).

One of the most impactful findings in the management of IgE-mediated CMA is the recent finding from Ireland indicating that BM can be introduced at home in infants using a milk ladder approach (7, 9). One study indicated that 65% of children safely consumed CM 12 months post randomization using a milk ladder approach, and 86% were safely consuming baked foods at 6 months post randomization (7). This review offers recommendations on facilitating safe use of milk ladders for clinical use in IgE-mediated CMA to improve future management of CMA.

## Reviewing the basis for ladders

CM contains a range of proteins of which 80% are casein proteins and 20% whey proteins. The allergenicity of these proteins are affected differently by different forms of heating, leading to the use of BM or milk ladders in the management of CMA. For example Bos d5 (beta-lactoglobulin) is found to be reduced by 99% with baking, whereas Bos d11 (b-casein) is reduced only by 30% (4, 24).

A food ladder is a stepwise progression from extensively heated to less heated food. Heating decreases the allergenicity of food proteins in egg and milk by degrading (altering) conformational epitopes so that the immune system has a reduced ability to recognize them (25). Heating has some but a limited effect on linear epitopes (25). Thus, it is assumed that progressing from extensively baked to less heated foods offers a progression from a less-allergenic to a more-allergenic form of the food protein. Food ladders also consider the amount of allergenic protein in each step of the ladder, which progressively increases as the ladder advances.

The first published ladder was created in 2013 for non-IgE mediated CMA (26) in the United Kingdom (UK) by Venter et al. This ladder initially contained 12 steps focusing on common British foods and was updated to a shortened ladder in 2017 that was more internationally focused regarding foods recommended (23). This ladder has been widely adopted for non-IgE-mediated CMA (27). Although initially created for non-IgE-mediated allergies, many providers also use ladders for progressive induction of tolerance at home for IgE-mediated allergies, especially to egg and CM (28). For example, one international survey found that as many as 60% of healthcare professionals responding to the survey used CM ladders for IgE-mediated food allergies (27).

There is evidence, although limited, demonstrating the development of tolerance via ladders. There have been recent publications regarding the use of home egg and CM ladders in Ireland, where pediatric allergy resources are limited, showing the safe use of a multi-step ladder. A significant number of participants achieved tolerance of egg or CM in all forms at the end of the study, even within the first year of life (28, 29). These studies were not controlled trials and included small sample sizes, limiting their generalizability.

Given that food ladders entail offering a child a known food allergen in the home, they come with inherent risk. Prior small-scale, non-randomized controlled trials (RCT) studies have reported their safe use, but the true risk of home-use of a food ladder has not been characterized (28), nor has it been described who may tolerate a ladder and who may not.

Additionally, home preparation of a ladder is not without risk, with the possibility that the amount of the allergenic protein differs from batch to batch of the same recipe or commercial food product. Further, the allergenic protein can even vary within a single serving, with the middle portion of the food being at higher risk for underbaking. Hindley et al. noted that in a BM muffin used in OFCs for CMA, baking partially denatured Bos d 11 (casein) at the periphery and had little effect on Bos d 11 in the remainder of the muffin. Bos d 5 (b-lactoglobulin) was more effectively denatured throughout the muffin (24). Thus, a ladder that could be safely used in IgE-mediated CMA would ideally have clear, simple instructions and have undergone some standardization in regard to the amount of food protein from batch to batch.

A published rostrum by Venter et al. (30) reviewed the current scientific basis for food ladders, their benefits and risks, and recommendations for the future. Possible benefits to using a ladder approach for IgE-mediated food allergy include (1) hastening of resolution of a food allergy (18), (2) increased diet diversity (31), (3) less healthcare utilization, (4) decreased cost, and (5) decreased patient burden (30). The rostrum also recommended standardization of food ladders regarding the allergenic protein content and cooking instructions for recipes, consideration of nutritional and health value of foods, acceptance of the food by a pediatric patient, and consideration of local/cultural eating practices. A review of the pros and cons of the use of ladders in IgE-mediated CMA is reviewed in Table 1.

**Table 1:** Pros/cons of milk ladders in IgE-mediated CMA and patient selection factors.

Milk ladders in IgE-mediated CMA	
Pros	Cons
A majority of CMA patients tolerate baked CM	Risk of anaphylaxis remains
Home-use decreases need for OFCs	Labor and resource intensive
Expansion of available foods in diet	Dependent on child’s acceptance of offered foods

Patient/family factors	
Good fit	Barriers
Tolerance of baked milk	History of anaphylaxis to baked milk
Motivated family	Uncontrolled co-morbid conditions, i.e., asthma
Family comfort managing allergic reactions in the home	Socioeconomic barriers, i.e., language barrier, limited financial resources
Young age	Older age

## Assessing ladders

When considering the various currently available CM ladders, which are examined individually in the following section, it is important to consider aspects related to the ladder itself; the patient/family in question; the healthcare system in which the patient exists; and the ladders impact on the patient’s nutrition, outside of allergen exposure.

### Ladder design

For use in clinical practice for IgE-mediated allergy, a ladder must offer a stepwise progression of CM protein content, with decreased denaturing as the ladder progresses, to serve the desired effect. The initial dose of CM protein must balance safety and efficiency, not adding unnecessary steps but being a low enough starting dose to be safely initiated in a majority of patients. Subsequent steps of the ladder should again follow reasonable increases in protein content. The most effective starting dose as well as the rate at which the dose should increase is an area that needs further exploration.

Foods in a single step should also contain a similar amount of CM protein (23, 26, 32, 33), which is often not the case in some currently available ladders (34, 35), which can have significant variability in the food choices on a single ladder step.

Given that the ladder is intended for home use, ladders should also provide clear, simple recipes for families to follow, given the significant variability in milk protein content in different variations of a food type, such as a muffin (23, 26, 32, 33). Unfortunately, some of the currently available ladders do not offer recipes but only list food types to be offered, i.e., muffins or pancakes (34, 35).

Ideally, as part of the design process of the ladder, the calculated milk protein content should be verified via lab quantification (32, 33). This has not been the case in many of the currently available ladders. The ladders that have taken this step demonstrate the need, as there is often discrepancies between the calculated and tested milk protein content. Further, the total milk protein content can differ compared to the milk component content, meaning the foods could be arranged in a different order depending on if total milk protein vs. a milk protein component progression is used as the goal (32).

Another consideration in ladder selection is nutritional content as well as palatability. Given that ladders are primarily intended for use in infants, toddlers and young children, the nutritional content is of supreme importance but also has to be balanced with the sometimes-limited palate of this age group. Ladders should strive to limit additions of “less-nutritious” ingredients, including refined sugar and provide nutrient-dense ingredients, such as fiber, as able (23, 33). However, they must also be palatable to be useful, given if the child refuses to eat the food regularly, it will not be able to offer its desired effect. Cultural appropriateness of the food items are also important, as well as the ease in which families can acquire the needed ingredients (32).

### Patient selection

Beyond the components of the ladder, consideration of patient-specific factors is also paramount for safe and successful use of a food ladder. Safety considerations are of highest importance, and it can be difficult to predict who may develop severe symptoms while stepping up a ladder. Prior reaction history to an allergenic food is not a strong indicator of future reactions (36). Further, modifying factors, such as illness, fatigue, exercise, or other poorly controlled atopic diseases (i.e., asthma), can lower a child’s tolerance and make day-to-day consumption of a food allergen at home not without continual risk (37). A recent pediatric death, partially attributed to an unstandardized approach to BM intake, highlights the need for more investigation of the safety and effectiveness of a food ladder for IgE-mediated food allergy (38).

Outside of the patient’s tolerance to the food allergen, family factors such as willingness and ability to procure and prepare the ladder foods must be considered. A myriad of socio-economic factors can make proper use of a ladder difficult, including but not limited to food costs, limited time, and language barriers.

The provider should also assess the family’s ability to respond to any allergic reaction that occurs and consider their ability to access emergency services, should that be required.

### Healthcare system

The healthcare system the patient resides in may alter the usability of a ladder, outside the availability of emergency medical services. Healthcare systems with limited subspecialty access, including pediatric allergists, may find ladders as a helpful alternative to observed OFCs to BM, which are resource and time intensive. As referenced above, there have been recent publications regarding the use of home egg and CM ladders in Ireland, where pediatric allergy resources are limited, showing the safe use of a multi-step ladder (28, 29). Limited healthcare resources also raises the question of who can safely prescribe use of a ladder. Prior work by our team has reported country-specific differences in the availability of allied health professionals (AHPs) such as Registered Dietitians (RDs), with some countries such as the UK having far more RDs available per patient and education regarding food avoidance and introduction often coming from these AHPs vs. a medical provider (20). There may be concern for the recommendation of ladders without direct consultation of a medical provider specialized in Allergy, but resource limitations in some regions of the world may necessitate relying on AHPs to administer ladders.

The healthcare cost of using a ladder should also be considered. It would likely cost less than an OFC to BM, but still requires subspeciality care with routine follow-up as well as coverage of emergency medications including epinephrine auto-injectors to be available at all times for patients utilizing a ladder approach.

The healthcare system and environment may also impact a provider’s comfort of prescribing use of a ladder, given there is inherent risk, and providers in countries with more litigious medicolegal environments may be hesitant to extensively recommend use of home ladders.

### Nutrition

Providers can also consider the impacts on nutrition and quality of life outside of allergen exposure when considering utilizing a ladder. It would be assumed that use of a ladder would broaden the foods available for a child to ingest, which would have a positive impact on their diet diversity as well as likely the quality of life of the child and their family given the decrease in dietary restrictions (31). This could also possibly result in improved growth parameters, as many food-allergic children having sub-optimal nutrient intake and growth due to their dietary restrictions (39). Many of these factors require further study to prove that such positive impacts truly do occur with use of a food ladder.

## Comparison of current milk ladders

**Figure 1:**
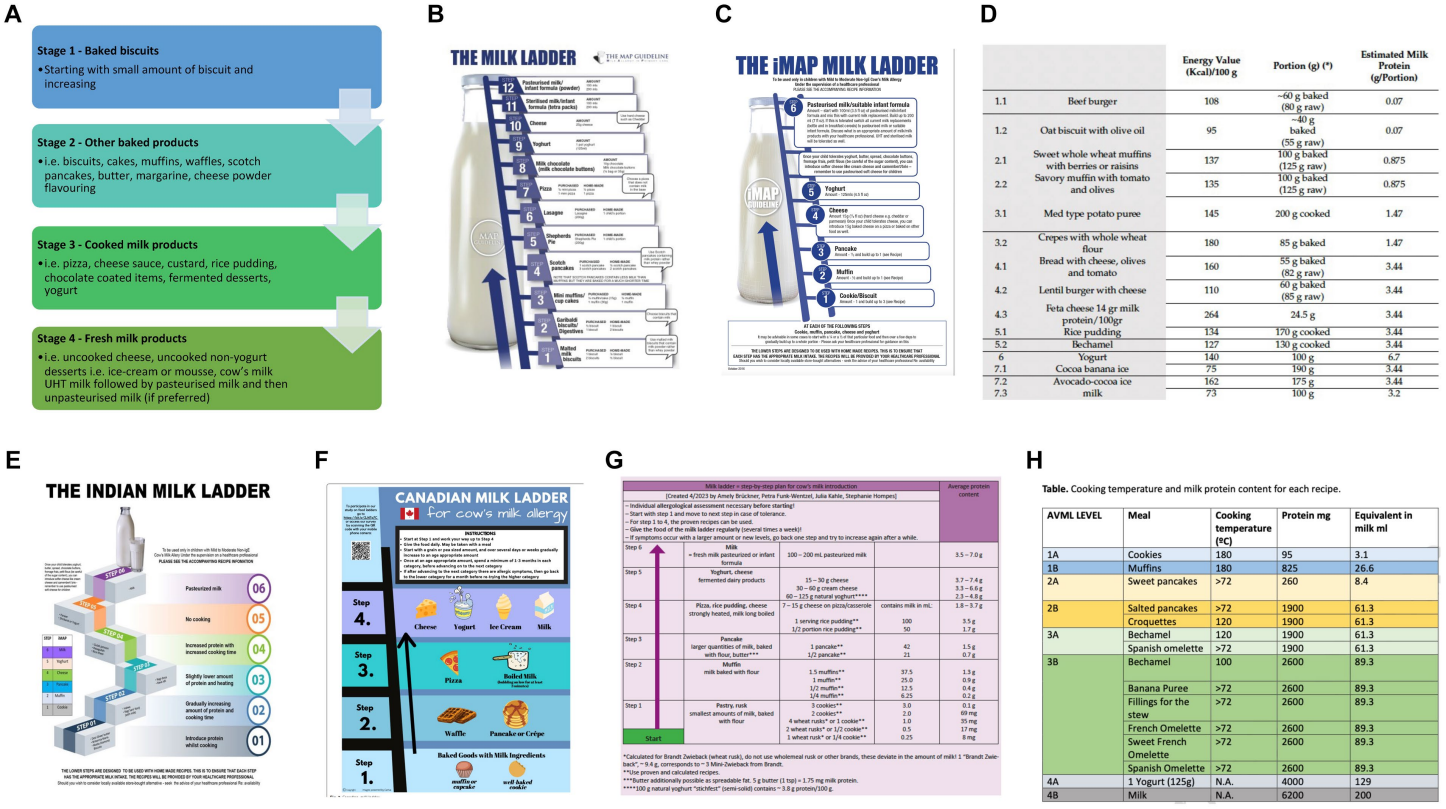
Current milk ladders **(A)** BSACI **(B)** MAP **(C)** iMAP **(D)** Mediterranean **(E)** Indian **(F)** Canadian **(G)** German **(H)** Spanish.

Multiple food ladders are currently available for use. Though many factors have been discussed above relating the safe use ladders, ultimately the safety of ladders depend on whether the steps are planned on calculated sequential increase of allergenicity, and ideally, if the allergenicity of the different steps have been tested (see Figure 1; Table 2).

**Table 2:** Comparison of currently available milk ladders.

# Steps	# Foods/step	Recipes included	Dose escalation	Starting/Ending dose	Measured Protein Content	Nutritional soundness	Culturally appropriate	Other Comments
BSACI (35)								
4	Multiple	No	Starts small but quickly escalated CM protein	Not listed	No	X	For British population	Foods in a single step are dissimilar in allergenicity
MAP (26)								
12	1	Yes	Moderate jump in steps (some steps subdivided into multiple steps)	95 mg/7.2 g	No	X	UK diet specific	Complex recipes
iMAP (23)								
6	1	Yes	Large jumps in steps (some steps subdivided into multiple steps)	35 mg/6.9 g	No	Yes	International	Simple recipes
Mediterranean milk ladder (33)								
7	1	Yes	Moderate jump in steps	70 mg/3.2 g	Yes – total protein, casein and beta-lactoglobulin	X	Mediterranean	Calculated and measured CM protein not always similar
Indian milk ladder (32)								
6	2–4	Yes	Moderate jump in steps	50 mg/8.68 g	Yes – total protein	High in sugar and fat – though recipes were adjusted to reduce sugar & fat content as able	Culturally relevant to India	Calculated and measured CM protein not always similar
Canadian milk ladder (34)								
4	2–4	No	Discrepancies in protein content in single steps	Not listed	No	X	Canadian foods	Simple
German milk ladder (40)								
6	1–3	Yes	Moderate jump in steps (each step is subdivided into multiple steps)	8 mg/7 g	No	X (some recipes adapted to contain less sugar)	German	Each step with progressive serving increases of the same food
Spanish milk ladder (41)								
4	1–6	Yes (not published currently)	Large jumps in some steps (each step is subdivided into multiple steps)	95 mg/6.2 g	No	Yes	Spanish	

## Discussion

Ladders offer unique aspects that make them a desirable method of allergen introduction in some children with CMA. However, ladders are not without risk and dependent on the particular patient and ladder in use. We offer the following recommendations for the favorable use of ladders.

Patient selection is of utmost importance in the safe use of ladders. Ladders can be readily utilized in children with non-IgE mediated allergy, excluding FPIES, for a gradual introduction of a previously avoided food (35). In the setting of IgE-mediated allergy, the patient ideally will have a history of prior mild reactions to CM and a higher prior tolerance level, although again prior reactions are not clear indications of any future reactions. The patient’s comorbid conditions including asthma must be well managed to prevent more severe potential reactions. No language or comprehension barriers should exist, and families should have the time and resources needed to use the ladder. Families should also have education on reaction management, should have emergency medications in the home and should have ready access to emergency services. Lastly, a younger age may be preferred as older patients may be prone to persistence of allergy (30).

Aspects of the ladder design also must be considered for successful use. Ladders should offer clear information on food allergen content. This should include calculation and ideally measurement of the allergenic protein content. The ladder should include similar items in terms of allergenic protein content in each step, with clear recipes specifying time and temperature of heating. The health and nutritional value of the food as part of the patient’s diet should be considered as well as the taste and acceptance of the food. Culturally appropriate ladders should be provided, and commercial options can be offered as able. There should also be clear guidance to families on how to offer each step and for how long prior to progressing, as well as instructions for safe dosing, i.e., when the child is in their normal state of health, in the home with a parent/guardian and access to emergency medications.

### Benefits beyond allergen introduction

There are benefits outside allergen introduction in the use of food ladders for IgE-mediated CMA. This includes nutritional aspects such as increased food introduction and potential expanded diet diversity and increased fiber intake.

For families utilizing a ladder, they may appreciate the decreased need for label reading and less concern about precautionary advisory labeling (31). There may be a subsequent reduction in food related anxiety (31). The expansion of the diet may also improve socialization and expand/normalize the child’s diet. There may be a financial benefit in a decreased need for observed OFCs, if the family would be expected to shoulder some of the financial cost of these challenges.

With the thought that ladders, with their gradual introduction of allergen, may promote tolerance, as well as their benefits outside of allergen introduction, they are a useful tool for providers to utilize in a carefully selected patient. Further studies both working on the creation of a ladder that meets all recommendations for safe use are needed, as well as studies that demonstrate their effectiveness in tolerance induction and their positive benefits outside of allergen introduction. However, while we await further investigation, ladders can be used judiciously in the properly selected patient with positive results. Future management of children with CMA may also include discussion around the immunomodulatory potential of the child’s dietary intake, which includes factors considered in the ladder such as sugar, fat and fiber intake.

## Data availability statement

The original contributions presented in the study are included in the article/supplementary material, further inquiries can be directed to the corresponding author.

## Author contributions

AH: Writing – original draft, Writing – review & editing. DF: Data curation, Writing – review & editing. CV: Writing – original draft, Writing – review & editing.

## Footnotes

^1^Waserman S, Bahna SL, Arasi S, Canani RB, Dupont C, Shamir R, et al. World allergy organization (WAO) diagnosis and rationale for action against Cow’s Milk allergy (DRACMA) guidelines update – IV clinical presentations: IgE-mediated & non IgE-mediated. *World Allergy Organ J.* (2023) Submitted.
