# Corrigendum: The future of cow's milk allergy – milk ladders in IgE-mediated food allergy

**DOI:** 10.3389/fnut.2025.1521516

**Published:** 2025-04-01

**Authors:** Allison Hicks, David Fleischer, Carina Venter

**Affiliations:** Section of Pediatric Allergy and Immunology, Children's Hospital Colorado, University of Colorado School of Medicine, Aurora, CO, United States

**Keywords:** food allergy, cow's milk allergy, nutrition, food ladders, pediatric

In the published article, there was an error in Table 2 as published. The starting and ending dose for the MAP and iMAP ladders were transposed. The starting dose of the MAP ladder is 95 mg and the starting dose of the iMAP ladder is 35 mg. The corrected Table 2 and its caption “Comparison of currently available milk ladders” appear below.

**Table 2:** Comparison of currently available milk ladders.

**# Steps**	**# Foods/ step**	**Recipes included**	**Dose escalation**	**Starting/ ending dose**	**Measured protein content**	**Nutritional soundness**	**Culturally appropriate**	**Other comments**
**BSACI (36)**								
4	Multiple	No	Starts small but quickly escalated CM protein	Not listed	No	X	For British population	Foods in a single step are dissimilar in allergenicity
**MAP (27)**								
12	1	Yes	Moderate jump in steps (some steps subdivided into multiple steps)	95 mg/7.2 g	No	X	UK diet specific	Complex recipes
**iMAP (24)**								
6	1	Yes	Large jumps in steps (some steps subdivided into multiple steps)	35 mg/6.9 g	No	Yes	International	Simple recipes
**Mediterranean milk ladder (34)**								
7	1	Yes	Moderate jump in steps	70 mg/3.2 g	Yes—total protein, casein and beta-lactoglobulin	X	Mediterranean	Calculated and measured CM protein not always similar
**Indian Milk Ladder (33)**								
6	2–4	Yes	Moderate jump in steps	50 mg/8.68 g	Yes—total protein	High in sugar and fat—though recipes were adjusted to reduce sugar & fat content as able	Culturally relevant to India	Calculated and measured CM protein not always similar
**Canadian milk ladder (35)**								
4	2–4	No	Discrepancies in protein content in single steps	Not listed	No	X	Canadian foods	Simple
**German milk ladder (41)**								
6	1–3	Yes	Moderate jump in steps (each step is subdivided into multiple steps)	8 mg/7 g	No	X (some recipes adapted to contain less sugar)	German	Each step with progressive serving increases of the same food
**Spanish milk ladder (42)**								
4	1–6	Yes (not published currently)	Large jumps in some steps (each step is subdivided into multiple steps)	95 mg/6.2 g	No	Yes	Spanish	

The authors apologize for this error and state that this does not change the scientific conclusions of the article in any way. The original article has been updated.

